# Dichotomisation of a continuous outcome and effect on meta-analyses: illustration of the distributional approach using the outcome birthweight

**DOI:** 10.1186/2046-4053-3-63

**Published:** 2014-06-12

**Authors:** Mercy Ofuya, Odile Sauzet, Janet L Peacock

**Affiliations:** 1 Division of Health and Social Care Research, King’s College London, 42 Weston Street, London SE1 3QD, UK; 2 AG Epidemiologie & International Public Health, Universitat Bielefeld, Bielefeld, Germany; 3 NIHR Biomedical Research Centre at Guy’s and St Thomas’ NHS Foundation Trust and King’s College London, London, UK

**Keywords:** Dichotomisation, Meta-analysis, Continuous outcome, Distributional method, Birthweight

## Abstract

**Background:**

Power and precision are greater in meta-analyses than individual study analyses. However, dichotomisation of continuous outcomes in certain studies poses a problem as estimates from primary studies can only be pooled if they have a common outcome. Meta-analyses may include pooled summaries of either or both the continuous and dichotomous forms, and potentially have a different combination of studies for each depending on whether the outcome was dichotomised in the primary studies or not. This dual-outcome issue can lead to loss of power and/or selection bias. In this study we aimed to illustrate how dichotomisation of a continuous outcome in primary studies may result in biased estimates of pooled risk and odds ratios in meta-analysis using secondary analyses of published meta-analyses with the outcome, birthweight, which is commonly analysed both as continuous, and dichotomous (low birthweight: birthweight < 2,500 g).

**Methods:**

Meta-analyses published in January 2010 - December 2011 were obtained using searches in PubMed, Embase, Web of Science, and Cochrane Database of Systematic Reviews with the outcome birthweight. We used a distributional method to estimate the pooled odds/risk ratio of low birthweight and its standard error as a function of the data reported in the primary studies of the included meta-analyses where accessible.

**Results:**

Seventy-six meta-analyses were identified. Thirty-seven percent (28/76) of the meta-analyses reported only the dichotomous form of the outcome while 26% (20/76) reported only the continuous form. In one meta-analysis (1/76), birthweight was analysed as continuous for one intervention and as binary for another and 36% (27/76) presented both dichotomous and continuous birthweight summaries. In meta-analyses with a continuous outcome, primary studies data were accessible in 39/48 and secondary analyses using the distributional approach provided consistent inferences for both the continuous and distributional estimates in 38/39.

**Conclusion:**

The distributional method applied in primary studies allows both a continuous and dichotomous outcome to be estimated providing consistent inferences. The use of this method in primary studies may restrict selective outcome bias in meta-analyses.

## Background

Meta-analyses of medical studies are conducted in order to synthesise research evidence on the subject of interest and provide an epidemiological evaluation of results from primary studies [1]. The use of meta-analysis allows us to quantify the pooled effect of an exposure variable, such as a risk factor or intervention, on an outcome of interest using the results from all available primary studies [2]. The precision and the statistical power of the hypothesis tested in a meta-analysis are usually higher than that of the primary studies due to the increase in the amount of data contributing to the overall pooled estimate [3].

Only primary studies with a common outcome can be pooled in a meta-analysis and so dichotomisation of continuous outcomes presents a difficulty over and above the loss of power [4], underestimation of effect size [5], and the need for larger samples [6,7] associated with the practice. When different cut-points for a particular continuous outcome have been used in primary studies, their results cannot be compared in a meta-analysis [4]. Pooling primary studies with the continuous and binary form of an outcome in separate meta-analyses [8], may lead to conflicting results and conclusions [8,9] due to loss of power and selection bias. More precisely, primary studies included in the calculation of pooled estimates may differ for the continuous and dichotomous form according to data presented in the separate reports and, therefore, a meta-analysis may not include all the primary studies carried out on a research question, leading to an incomplete and potentially biased summary of the evidence. Further, information from the same primary study may be used in both meta-analyses, thus making the results repetitive and not necessarily confirmatory [9].

Peacock *et al*. [10] have previously described a distributional method for use in primary studies which permits researchers to present both the comparison of means and comparison of proportions. This method involves transforming the difference in means between two groups, into a comparison of proportions of subjects that fall below (or above) a threshold of interest, to give a ‘distributional estimate’ expressed as a difference in proportions, risk ratio (RR) or odds ratio (OR). The standard error for the distributional estimate is derived as a function of the means and standard deviations of the sample using the delta method and so inferences drawn from the comparison of proportions reflect inferences about the comparison of means. The purpose of this study was to use the distributional method described above to illustrate how dichotomisation of a continuous outcome in primary studies may result in biased estimates of pooled RRs and ORs in meta-analysis particularly when either outcome includes only a subset of the available primary studies. To do this, we considered an outcome that is commonly reported as dichotomous and/or continuous, birthweight (analysed as continuous (g), or as dichotomous (low birthweight (LBW): < 2,500 g). This threshold (2,500 g) is clinically relevant both in clinical trials and epidemiology spanning various areas of health research.

## Methods

### Search strategy

Searches of electronic databases were conducted in PubMed, Embase, Web of Science and the Cochrane Database of Systematic Reviews from January 2010 to December 2011 using search terms ‘birthweight’ OR ‘birth weight’. The search was limited to meta-analyses and human studies in PubMed and Embase. The references of the papers that met the inclusion criteria were searched for additional studies.

**Figure 1:**
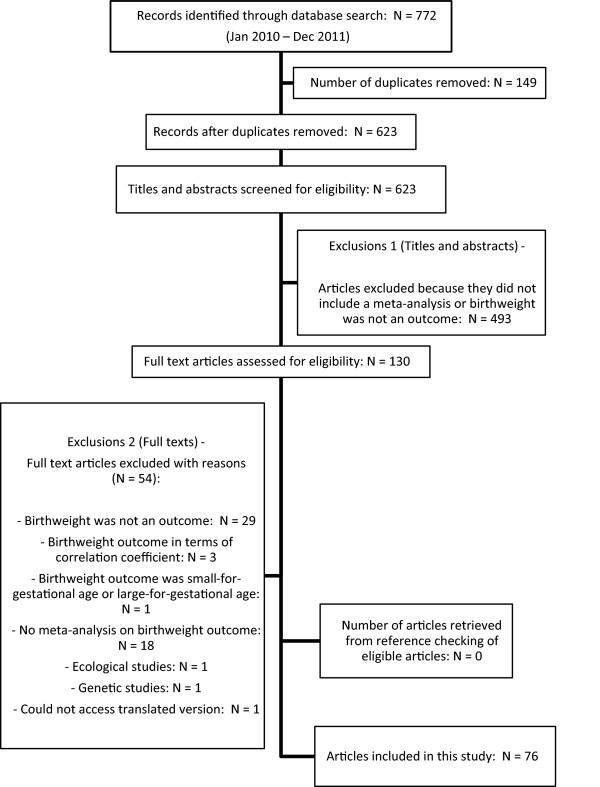
Flow diagram of search process for meta-analyses included in this study.

Meta-analyses in which birthweight was an outcome variable (either primary or secondary) were eligible. Meta-analyses were excluded if birthweight was a risk factor and not an outcome and if the systematic review did not include a meta-analysis on a birthweight outcome. If the birthweight outcome was ‘small and/or large for gestational age’ or presented in terms of correlation coefficient, the meta-analyses were excluded. Other exclusion criteria were genetic and ecological studies. Titles and abstracts were screened and the full texts of studies which met the eligibility criteria were retrieved. The flowchart showing the search strategy is presented in Figure 1 in accordance with the Preferred Reporting Items for Systematic Reviews and Meta-Analyses (PRISMA) statement [11].

### Illustrative analyses

A secondary analysis was performed in order to illustrate the consequences of dichotomisation. The distributional method was used to obtain distributional RR/ORs for each of the primary studies included in each meta-analysis using the reported sample means and standard deviations. These distributional estimates for LBW were then pooled to obtain a summary distributional RR with confidence intervals (CIs) using either the fixed or random effects model as appropriate. We refer to this pooled estimate as the ‘pooled distributional estimate’. This process was undertaken using meta-analyses for which the means and standard deviations of the birthweight data in the pooled primary studies could be accessed. The various steps for the application of the distributional method in obtaining distributional risk and ORs have been set out in another paper [10] and a Stata ado-file is available (http://wwwhomes.uni-bielefeld.de/osauzet/distributional.htm).

For this illustrative study, we assume that birthweight follows a normal distribution as required by the distributional method [10].

All the analyses were performed using Stata version 12.0 [12].

## Results

### Meta-analyses included in study

**Figure 2:**
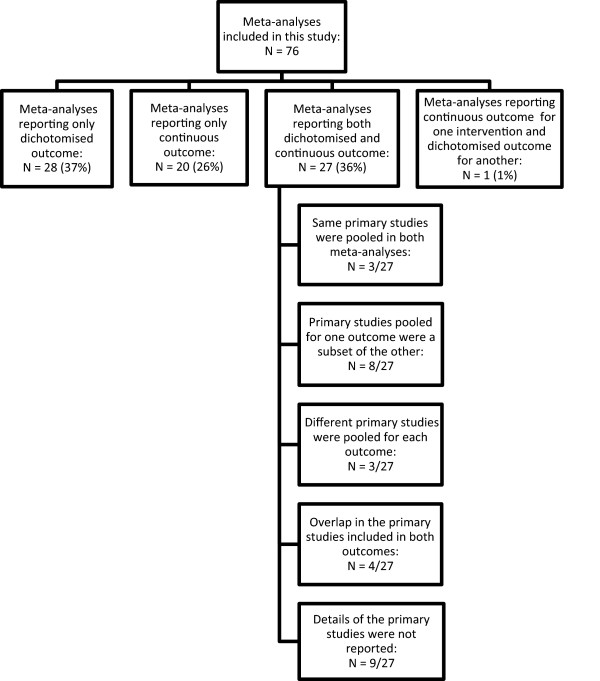
Flow diagram showing details of meta-analyses included in this study.

A total of 772 papers were retrieved from the search and of these, 76 published meta-analyses which met the inclusion criteria were included in this study. Fifty-two (52/76) of the meta-analyses included in this study combined randomised controlled trials, and in 24/76, observational studies were pooled. Details of the meta-analyses included in this study can be found in an additional file [see Additional file 1].Thirty-seven percent (28/76) of the meta-analyses reported only the dichotomous form of the outcome while 26% (20/76) reported only the continuous form. In 1/76 meta-analysis, birthweight was analysed as continuous for one intervention and as binary for another. Thirty-six percent (27/76) of the meta-analyses presented both binary and continuous forms of the variable. Among these, 7/27 reported results that were statistically significant for one outcome and not for the other. The number of meta-analyses reporting either dichotomous or continuous outcomes is presented in a flow diagram (see Figure 2). There was no discussion related to the presentation of two separate meta-analyses for both forms of the outcome in any of the 76 meta-analyses papers included in this study.

### Using the distributional method in secondary meta-analysis

Tables 1 and 2 show the results of the secondary meta-analyses performed to illustrate how the results might have looked had the distributional method been used to give means and proportions in all primary studies. Secondary analyses were performed using data reported in meta-analyses (N = 39/76) where primary study means and standard deviations were reported and could be accessed (that is meta-analyses reporting a continuous outcome: N = 21/39; meta-analyses reporting both continuous and dichotomous: N = 18/39).

**Table 1:** Secondary analyses in meta-analyses reporting only birthweight mean difference outcome (N = 21)

**Meta-analysis**			**Published**		**Distributional estimates for low birthweight**		
	**Number of studies**	**Pooled sample size**	**Mean difference (g) (95% CI)**	***P*-value**	**Distributional RR (95% CI)**	***P*-value**	**Comments**
Abou El Senoun 2010 [13]	1	55	−170 (−558, 218)	0.39	1.13 (0.85, 1.51)	0.39	
Alfirevic 2010 [14]	7	3,887	28 (−10, 66)	0.15	0.96 (0.89, 1.04)	0.28	
Alfirevic 2010 [15]	2	5,914	−18 (−42, 7)	0.16	1.08 (0.97, 1.19)	0.15	
Begley 2010 [16]	2	3,207	−77 (−109, −45)	< 0.01	1.49 (1.27, 1.77)	< 0.01	
Bevilacqua 2010 [17]	7	5,372	−83 (−124, −42)	< 0.01	1.04 (1.02, 1.08)	< 0.01	
Blanco 2011 [18]	2	786	113 (−45, 271)	0.16	0.74 (0.48, 1.16)	0.19	
Buchanan 2010 [19]	7	692	−12 (−91, 67)	0.76	1.00 (0.97, 1.03)	0.87	
Coleman 2010 [20]	3	614	158 (−53, 370)	0.14	0.65 (0.36, 1.18)	0.16	
Crowther 2011 [21]	9	5,626	−76 (−118, −34)	< 0.01	1.02 (1.01,1.03)	0.01	
Dhulkotia 2010 [22]	6	1,388	24 (−36, 83)	0.44	0.93 (0.77, 1.13)	0.48	
Gebreselassie 2011 [23]	17	6,208	39 (−7, 85)	0.09	0.88 (0.76, 1.02)	0.08	
Imdad 2011 [24]	13	4,189	60 (33, 87)	< 0.01	0.79 (0.71, 0.87)	< 0.01	
Lassi 2010 [25]	2	1,050	11 (−39, 62)	0.66	0.98 (0.84, 1.14)	0.78	
Mackeen 2011 [26]	2	117	159 (−44, 361)	0.13	0.97 (0.93, 1.02)	0.19	
Mak 2010 [27]	4	251	8.33 (−143, 159)	0.91	0.99 (0.62, 1.58)	0.96	4/6 primary studies were accessible
Middleton 2010 [28]	2	159	−3 (−180, 175)	0.98	0.99 (0.53, 1.90)	0.99	
Nabhan 2011 [29]	1	125	−100 (−364, 164)	0.46	1.18 (0.78, 1.80)	0.46	
Quinlivan 2011 [30]	4	537	8.5 (−85, 102)	0.86	0.97 (0.67, 1.41)	0.88	
Rumbold 2011 [31]	5	7,497	6.1 (−17, 29)	0.61	0.99 (0.91, 1.08)	0.84	
Stampalija 2010 [32]	1	3,133	−34 (−69, 0.63)	0.05	1.15 (0.99, 1.33)	0.05	
Vazquez 2011^a^[33]	1	128	−461 (−608, −314)	< 0.01	4.91 (2.88, 8.37)	< 0.01	Data from meta-analysis where outcome was analysed as continuous for one intervention and as binary for another.

^a^Meta-analysis where outcome was analysed as continuous for one intervention and as binary for another; CI: confidence interval; RR: risk ratio.

**Table 2:** Secondary analyses for meta-analyses reporting both continuous and dichotomous outcomes (N = 18)

**Meta-analysis**	**Published data**						**Distributional estimates for low birthweight**			
	**Number of studies (sample size)**	**Mean difference (g) (95% CI)**	***P*-value**	**Number of studies (sample size)**	**RR (95% CI)**	***P*-value**	**Number of studies (sample size)**	**Distributional RR (95% CI)**	***P*-value**	**Comments**
Bupassiri 2011 [34]	21 (8,319)	65 (16, 114)	0.01	5 (13,638)	0.83 (0.63, 1.09)	0.18	21 (8,319)	0.72 (0.58, 0.89)	< 0.01	
Crowther 2010 [35]	4 (417)	75 (−17, 167)	0.11	7 (1,452)	0.84 (0.68, 1.04)	0.12	4 (417)	0.99 (0.88, 1.06)	0.42	
Dodd 2010 [36]	2 (282)	−75 (−210, 61)	0.28	1 (49)	0.41 (0.04, 4.20)	0.45	2 (282)	1.33 (0.78, 2.26)	0.29	2/3 primary studies of mean birthweight outcome accessed
Gouin 2011 [37]	18 (6,855)	−441 (−532, −350)	< 0.01	19 (38,796)	2.86 (2.36, 3.48)	< 0.01	18 (6,855)	2.76 (2.12, 3.45)	0.01	
Gülmezoglu 2011 [38]	1 (208)	−100 (−240, 40)	0.16	1 (604)	1.38 (0.92, 2.06)	0.12	1 (208)	1.40 (0.87, 2.24)	0.16	
Kawai 2011 [39]	13 (35,015)	45 (28, 62)	< 0.01	13 (35,015)	0.92 (0.83, 1.02)	0.09	13 (35,015)	0.82 (0.75, 0.91)	< 0.01	13/15 primary studies accessed
Kenyon 2010 [40]	13 (6,480)	49 (14, 85)	0.01	2 (4,876)	1.00 (0.96, 1.04)	0.94	13 (6,480)	0.99 (0.99, 1.00)	0.53	
Ladhani 2011 [41]	4 (880)	−279 (−485, −74)	0.01	2 (26,026)	3.28 (2.25, 4.78)	< 0.01	4 (880)	2.41 (1.42, 4.09)	< 0.01	
Lamont 2011 [42]	1 (485)	−12 (−128, 104)	0.89	2 (876)	0.96 (0.62, 1.47)	0.83	1 (485)	1.03 (0.77, 1.38)	0.84	
Mathanga 2011 [43]	2 (640)	121 (27, 214)	0.01	2 (624)	0.80 (0.54, 1.18)	0.25	2 (640)	0.75 (0.60, 0.94)	0.01	
McDonald 2010 [44]	9 (5,225)	−120 (−248, 6.8)	0.06	9 (5,225)	0.92 (0.72, 1.16)	0.46	9 (5,225)	1.12 (0.99, 1.26)	0.07	9/10 primary studies accessed
Murphy 2011 [45]	8 (179,589)	−121 (−199, −43)	< 0.01	12 (1,110,176)	1.45 (1.21, 1.73)	< 0.01	8 (179,589)	1.46 (1.10, 1.94)	0.01	
Reveiz 2011 [46]	3 (237)	15 (−111, 142)	0.81	1 (100)	Not estimated	NA	3 (237)	0.95 (0.59, 1.52)	0.83	Zero cases of LBW in both treatment arms of primary study
Salmasi 2010 [47]	44 (71,663)	13 (−105, 131)	0.83	18 (40,790)	1.09 (0.88, 1.35)	0.44	44 (71,663)	0.98 (0.77, 1.23)	0.85	18/19 primary studies of LBW outcome accessed
Whitworth 2010 [48]	5 (23,213)	11 (−20, 41)	0.49	8 (19,337)	1.04 (0.82, 1.33)	0.73	5 (23,213)	0.97 (0.87, 1.08)	0.56	
Wiysonge 2011 [49]	3 (1,809)	68 (19, 118)	0.01	4 (2,606)	0.83 (0.68, 1.01)	0.07	3 (1,809)	0.84 (0.74, 0.95)	0.01	
	**Number of studies (sample size)**	**Mean difference (g) (95% CI)**	***P*-value**	**Number of studies (sample size)**	**OR (95% CI)**	***P-*** **value**	**Number of studies (sample size)**	**Distributional OR (95% CI)**	***P*-value**	
Pope 2010 [50]	5 (13,955)	100 (73, 128)	< 0.01	8 (Unclear)	1.38 (1.25, 1.52)	< 0.01	5 (13,955)	0.81 (0.76, 0.86)	< 0.01	Pooled published pre-calculated estimates for LBW outcome (that is Log(OR) and SE)
Salvig 2010^a^[51]										
(Fixed effects model)	4 (1,187)	66 (1.6, 131)	0.04	3 (785)	0.98 (0.66, 1.46)	0.93	4 (1,187)	0.81 (0.65, 1.01)	0.07	
Salvig 2010^a^[51]										
(Random effects model)	4 (1,187)	68 (−75, 212)	0.35	3 (785)	0.95 (0.49, 1.85)	0.88	4 (1,187)	0.79 (0.48, 1.29)	0.34	

CI: confidence interval; LBW: low birthweight; NA: not applicable; OR: odds ratio; RR: risk ratio; SE: standard error; ^a^Fixed effects model used in published meta-analysis but there was significant heterogeneity between studies (*P* = 0.003) so secondary analysis was repeated here using the random effects model.

For the meta-analyses reporting only the mean difference outcome (N = 21/39) for an intervention/exposure, secondary analyses provide distributional estimates for low birthweight that reflect those of the mean differences (see Table 1).

Where both forms of outcome were reported, the number of pooled primary studies for each differed in 16/18 meta-analyses, so the results for different outcomes were based on different subsets of the available data (see Table 2). In 2/18 [39,44], the same studies were combined for each outcome and although the distributional RR were similar to those of the published RR, the CIs were narrower with inferences consistent with those of the mean differences.

The distributional estimates provided similar inferences to the mean difference outcomes in 17/18 meta-analyses (see Table 2) confirming that the distributional estimates are valid. For 1/18 meta-analysis [40], where the results of the distributional estimates were not consistent with that of the mean difference outcome (see Table 2), many of its primary studies were very small with very low means and so the distributional method would not be recommended [10].

Secondary analyses could not be performed on 28/76 meta-analyses which reported only the dichotomous form of the outcome. In a further 9/76 meta-analyses reporting both dichotomous and continuous forms of the birthweight outcome, details of the pooled primary studies’ data could not be accessed and the reasons are outlined in an additional file [see Additional file 2].

## Discussion

The aim of this study was to illustrate how dichotomisation of a continuous outcome in primary studies may result in biased estimates of pooled RRs and ORs in meta-analysis, using published meta-analyses reporting the birthweight outcome as an example. There is a difficulty in comparing results on the basis of statistical significance; a comparison of means will be more powerful than a comparison of proportions below a cut-point in the same datasets and, therefore, the former are more likely to be statistically significant. This is one reason why using a distributional method in primary studies to estimate proportions below a cut-point carries an advantage in that estimates of mean differences are comparable to estimates based on comparison of proportions [10].

Researchers commonly dichotomise continuous data such as birthweight as it may be difficult to interpret differences in means, but a difference in percentage of low birthweight may be more meaningful. When a continuous outcome is dichotomised in some primary studies but not others, this may cause difficulties for the meta-analyst. The process of selecting primary studies for inclusion in a meta-analysis may include deciding between studies reporting the continuous or the binary form of an outcome. In such cases, the set of studies reporting the continuous outcome may be different from those reporting the binary form. Where a different set of studies are combined for each of the two outcomes, there is the possibility of biased results. This is illustrated in Table 2 where the number of studies and/or sample sizes are very different for the two outcomes in 16/18 meta-analyses. For these meta-analyses, although the precision of the distributional estimates gave similar inferences as the mean differences, they were not comparable with those of the published LBW outcome because they are based on different sets of data.

We do not imply that the distributional approach gives the more accurate result in our illustration as the limited availability of data in published papers prevented its application for all studies. The best option is for primary studies to report both forms for birthweight as other researchers may wish to see them.

Several methods have been developed for combining individual studies reporting continuous and binary outcomes in one meta-analysis to obtain one summary measure in meta-analysis [8,9,52]. Whitehead *et al*. [8] obtained a summary log-odds ratio while other authors [9,52] have recommended converting the estimates from individual studies to effect sizes and then combining these. These methods are helpful in allowing all studies to be pooled but do not overcome the problem of the loss of power when dichotomising.

We have used the distributional method in secondary meta-analysis to demonstrate how dichotomisation in primary studies may result in inconsistent estimates in the context of meta-analyses. We are not advocating the distributional method as a tool for meta-analysts who are using aggregate data, but rather wish to highlight its usefulness in primary studies.

## Conclusions

Researchers who wish to dichotomise continuous outcomes in primary studies may consider using the distributional approach to obtain the difference in proportions or RR/OR to present alongside differences in means. Where this has not been done, and if the individual study outcomes follow a normal distribution and means, standard deviations, sample sizes are given, the meta-analyst can compute distributional estimates for use in pooled summaries. In this way, meta-analyses will be less subject to selective outcome bias.

## Abbreviations

CI: confidence interval; LBW: low birthweight; OR: odds ratio; RR: risk ratio; SE: standard error; PRISMA: Preferred Reporting Items for Systematic Reviews and Meta-Analyses; NA: not applicable.

## Competing interests

The authors declare that they have no competing interests.

## Authors’ contributions

MO: design, data extraction, analyses, manuscript writing and final approval of manuscript; OS: design, manuscript writing, critical revision and final approval of manuscript; JP: design, manuscript writing, critical revision and final approval of manuscript. All authors read and approved the final manuscript.

## Supplementary Material

Additional file 1: Table S1.1 Details of meta-analyses included in this study. Click here for file

Additional file 2: Table S1 Meta-analyses for which secondary analyses could not be performed with reasons. Click here for file
