# Supplementary material for: Dichotomisation of a continuous outcome and effect on meta-analyses: illustration of the distributional approach using the outcome birthweight
Source: Syst Rev. 2014 Jun 12;3:63. doi: 10.1186/2046-4053-3-63 (PMC4063432; doi:10.1186/2046-4053-3-63)
Supplement: Additional file 1: Table S1.1 — Details of meta-analyses included in this study. [file 2046-4053-3-63-S1.pdf]

Additional file 1

Table 1.1 Details of meta-analyses included in this study

Study	Comparison	Study design	Number of studies by outcome	Pooled sample size by outcome
**Abou El Senoun et al., 2010[1]**	Planned home versus hospital care for preterm pre-labour rupture of the membranes (PPROM) prior to 37 weeks' gestation.	RCT	BWMD: 1	BWMD: 55
**Akl et al., 2010[2]**	Effects of water pipe tobacco smoking on pregnancy outcomes.	Cohort and case control	LBW: 3	LBW: 9557
**Alexander et al., 2010[3]**	Systematic digital cervical examination versus no examination unless medically indicated.	RCT	LBW: 1<br>VLBW: 1	LBW: 5371<br>VLBW: 5371
**Alfirevic et al., 2010[4]**	Doppler ultrasound versus no doppler ultrasound in high risk pregnancies.	RCT	BWMD: 7	BWMD: 3887
**Alfirevic et al., 2010[5]**	All routine doppler ultrasound versus no Doppler ultrasound in normal pregnancies.	RCT	BWMD: 2	BWMD: 5914
**Begley et al., 2010[6]**	Active versus expectant management of 3rd stage of labour.	RCT	BWMD: 2	BWMD: 3207
**Bevilacqua et al., 2010[7]**	Multiple courses versus single course of antenatal corticosteroids.	RCT	BWMD: 7	BWMD: 5372
**Blanco et al., 2011[8]**	Fetal outcomes in pregnant women with type 1 diabetes mellitus treated with lispro versus regular insulin.	Cohort	BWMD: 2	BWMD: 786
**Bonzini et al., 2011[9]**	Shift work and risk of having a low birthweight at delivery.	Cohort and cross sectional	LBW: 6	LBW: 41750

RCT: Randomised controlled trials; LBW: Low birthweight; VLBW: Very low birthweight; BWMD: Birthweight mean difference (in grams)

References for the meta-analysis papers in the table are listed after the table and are numbered sequentially starting at [1].
Reference numbers for the additional files are separate from the overall reference list for this study.

Table 1.2 Table 1.1 Details of meta-analyses included in this study (continued)

Study	Comparison	Study design	Number of studies by outcome	Pooled sample size by outcome
**Buchanan et al., 2010[10]**	Planned early birth versus expectant management for women with preterm pre-labour rupture of membranes prior to 37weeks' gestation.	RCT	BWMD: 7	BWMD: 692
**Buppasiri et al., 2011[11]**	Effect of calcium supplementation versus placebo on birth outcomes.	RCT	BWMD: 21<br>LBW: 5	BWMD: 8319<br>LBW: 13638
**Coleman 2010[12]**	Efficacy and safety of nicotine replacement therapy (NRT) with or without behavioural support when used to support smoking cessation in pregnancy.	RCT	BWMD: 3	BWMD: 614
**Crowther and Han 2010[13]**	Hospitalisation for bed rest in women with a multiple pregnancy.	RCT	BWMD: 4<br>LBW:7<br>VLBW:7	BWMD:417<br>LBW:1452<br>VLBW:1452
**Crowther et al., 2011[14]**	Repeat doses of corticosteroids versus single course in women at risk of preterm birth for preventing neonatal respiratory disease.	RCT	BWMD:9<br>BW Z-scores:2<br>BW MoM: 1	BWMD: 5626<br>BW Z-scores: 1256<br>BW MoM: 590
**De -Regil et al., 2010[15]**	Supplementation with folic acid plus other micronutrients versus other micronutrients (without folic acid) in pregnant women.	RCT	LBW: 1	LBW: 186
**Dhulkotia et al., 2010[16]**	Effects of oral hypoglycaemic agents with insulin in achieving glycemic control and the perinatal outcomes in gestational diabetes mellitus.	RCT	BWMD: 6	BWMD: 1388

RCT: Randomised controlled trials; LBW: Low birthweight; VLBW: Very low birthweight; BWMD: Birthweight mean difference (in grams); MoM: Multiples of the median; BW Z-scores: Birthweight z-scores.

References for the meta-analysis papers in the table are listed after the table and are numbered sequentially starting at [1].
Reference numbers for the additional files are separate from the overall reference list for this study.

Table 1.3 Table 1.1 Details of meta-analyses included in this study (continued)

Study	Comparison	Study design	Number of studies by outcome	Pooled sample size by outcome
**Dodd et al., 2010[17]**	Effects of antenatal dietary or lifestyle interventions in pregnant women who are overweight or obese.	RCT	BWMD: 3<br>LBW: 1<br>HBW: 1	BW:367<br>LBW: 49<br>HBW:49
**Dodd et al., 2010[18]**	Heparin (alone or with other medication) versus no treatment in women considered at risk of placental dysfunction.	RCT	LBW: 1	LBW: 110
**Dowswell et al., 2010 [19]**	Reduced number of antenatal care visits/goal oriented versus standard antenatal care visits.	RCT	LBW: 6	LBW:NS
**Eisele et al., 2010[20]**	Efficacy of IPTp and ITNs used during first or second pregnancy for preventing adverse birth outcomes.	CRCT	LBW: 5	LBW: 3360
**Finkelsztejn et al., 2011[21]**	Risk of having a baby with low birthweight in pregnant women with multiple sclerosis.	Cohort	LBW: 8	LBW: 2002
**Gebreselassie and Gashe, 2011[22]**	Effect of prenatal zinc supplementation on birthweight.	RCT	SMD: 17	SMD: 6208
**George et al., 2011[23]**	Assessed the potential of periodontal treatment during pregnancy in reducing low birthweight incidence	RCT	LBW:7	LBW: 4105
**Gouin et al., 2011[24]**	Effect of maternal antenatal cocaine exposure on perinatal outcomes.	Cohort and case control	BWMD: 18<br>LBW: 19	BWMD: 6855<br>LBW: 38796

RCT: Randomised controlled trials; CRCT: Cluster randomized controlled trials; LBW: Low birthweight; VLBW: Very low birthweight; BWMD: Birthweight mean difference (in grams); HBW: High birthweight; SMD: Standardised mean difference.

References for the meta-analysis papers in the table are listed after the table and are numbered sequentially starting at [1].
Reference numbers for the additional files are separate from the overall reference list for this study.

Table 1.4 Table 1.1 Details of meta-analyses included in this study (continued)

Study	Comparison	Study design	Number of studies by outcome	Pooled sample size by outcome
**Grellier et al., 2010 [25]**	Association of total trihalomethane exposure in the third trimester and adverse birth outcomes.	Observational	LBW: 4<br>TLBW: 4	LBW:1002928<br>TLBW: 38893
**Grote et al., 2010[26]**	Antenatal depression and risk of low birthweight.	Observational	LBW: 11	LBW: 13544
**Gülmezoglu and Azhar, 2010 [27]**	Effectiveness of metronidazole for treatment of trichomoniasis in pregnant women.	RCT	BWMD:1<br>LBW:1	BWMD:208<br>LBW: 604
**Haider et al., 2011[28]**	Impact of multiple micronutrient supplements during pregnancy with standard iron-folate supplements on birth outcomes.	RCT	LBW: 1	LBW: 950
**Han et al.,2011[29]**	Effect of low gestational weight gain on adverse birth outcomes.	Case control and cohort	BWMD: 3<br>LBW:13<br>MLBW:1<br>VLBW:1	BWMD: Unclear<br>LBW: 3187619<br>MLBW: Unclear<br>VLBW: Unclear
**Han et al., 2011[30]**	Relationship between maternal underweight and low birth weight in singleton pregnancies.	Case control and cohort	BWMD: 8<br>LBW: 24<br>TLBW: 5<br>MLBW:1<br>VLBW: 2	BWMD: NS<br>LBW:190308<br>TLBW: 7307<br>MLBW: NS<br>VLBW: NS

RCT: Randomised controlled trials; LBW: Low birthweight; VLBW: Very low birthweight; MLBW: Moderately low birthweight; TLBW: Term low birthweight; BWMD: Birthweight mean difference (in grams); SMD: Standardised mean difference; NS: Not stated

References for the meta-analysis papers in the table are listed after the table and are numbered sequentially starting at [1].
Reference numbers for the additional files are separate from the overall reference list for this study.

Table 1.5 Table 1.1 Details of meta-analyses included in this study (continued)

**Study**	**Comparison**	**Study design**	**Number of studies by outcome**	**Pooled sample size by outcome**
**Hodnett et al., 2010[31]**	Effects of programs offering additional social support for pregnant women at risk of giving birth to low birthweight babies.	RCT	LBW: 11	LBW: 8681
**Hofmeyr et al., 2010[32]**	Effects of calcium supplementation during pregnancy on hypertensive disorders of pregnancy and related maternal and child outcomes.	RCT	LBW: 9	LBW: 14883
**Imdad and Bhutta 2011[33]**	Effect of balanced protein energy supplementation during pregnancy on birth outcomes.	RCT	BWMD: 13	BWMD: 4189
**Imdad et al., 2011[34]**	Effect of calcium supplementation during pregnancy in reducing risk of developing gestational hypertensive disorders on birth outcomes.	RCT	LBW: 3	LBW: 9498
**Kawai et al., 2011[35]**	Effect of supplementation with multiple micronutrients versus iron and folic acid on pregnancy outcomes.	RCT	BWMD: 15<br>LBW: 15	BWMD: 64244<br>LBW: 64244
**Kenyon et al., 2010[36]**	Effect of antibiotic treatment for preterm rupture of the membrane on birth outcomes.	RCT	BWMD:12<br>LBW:2	BWMD:6374<br>LBW: 4876
**Ladhani 2011[37]**	Effect of amphetamine exposure on birthweight and the rate of LBW infants.	Cohort	BWMD:4<br>LBW:2	BWMD:880<br>LBW:26026

RCT: Randomised controlled trials; LBW: Low birthweight; VLBW: Very low birthweight; BWMD: Birthweight mean difference (in grams).

References for the meta-analysis papers in the table are listed after the table and are numbered sequentially starting at [1].
Reference numbers for the additional files are separate from the overall reference list for this study.

Table 1.6 Table 1.1 Details of meta-analyses included in this study (continued)

**Study**	**Comparison**	**Study design**	**Number of studies by outcome**	**Pooled sample size by outcome**
**Lamont et al., 2011[38]**	Effect of clindamycin on birth outcomes.	RCT	BWMD:1<br>LBW:2<br>VLBW:2	BWMD:485<br>LBW:876<br>VLBW:876
**Lassi et al., 2010[39]**	Effectiveness of community-based intervention packages in improving neonatal outcomes.	RCT	BWMD: 2	BWMD: 1050
**Mackeen et al., 2011[40]**	Tocolytic therapy versus no treatment in women with preterm premature rupture of membranes.	RCT	BWMD: 2	BWMD: 117
**Mak et al., 2010[41]**	Combination of heparin and aspirin versus aspirin alone in enhancing live births in patients with recurrent pregnancy loss and positive anti-phospholipid antibodies.	RCT	SMD: 6	SMD: 334
**Matevosyan 2011[42]**	Effect of periodontal disease on pregnancy outcomes.	RCT and Case control	LBW: Unclear	LBW: Unclear
**Mathanga et al., 2011[43]**	Compared the effects of monthly sulfadoxine pyrimethamine (SP) to standard 2-dose SP on birth outcomes.	RCT	BWMD:2<br>LBW:2	BWMD:640<br>LBW:624
**Mcdonald et al., 2010[44]**	Association between maternal overweight and obesity (in mothers with singleton pregnancies) with preterm birth and low birthweight.	RCT	BWMD: 9<br>LBW:28	BWMD: NS<br>LBW: 293762

RCT: Randomised controlled trials; LBW: Low birthweight; VLBW: Very low birthweight; BWMD: Birthweight mean difference (in grams); SMD: Standardised mean difference; NS: Not stated.

References for the meta-analysis papers in the table are listed after the table and are numbered sequentially starting at [1].
Reference numbers for the additional files are separate from the overall reference list for this study.

Table 1.7 Table 1.1 Details of meta-analyses included in this study (continued)

Study	Comparison	Study design	Number of studies by outcome	Pooled sample size by outcome
**Mcdonald et al., 2010[45]**	Risks of adverse birth outcomes in twins conceived through in vitro fertilization (IVF) with IVF/intracytoplasmic sperm injection (ICSI) versus spontaneously-conceived twins.	Cohort and case-control	BWMD:10<br>LBW: 10<br>VLBW: 7<br>ELBW:2	BWMD: Unclear<br>LBW: Unclear<br>VLBW: Unclear<br>ELBW: Unclear
**Mclernon et al., 2010[46]**	Effectiveness of elective single embryo transfer versus double embryo transfer on birth outcomes.	RCT	BWMD: NS<br>LBW: NS	BWMD: NS<br>LBW: 465
**Metcalfe et al., 2011[47]**	Impact of neighbourhood income on low birthweight.	Cohort	LBW: 6	LBW: NS
**Middleton et al., 2010[48]**	Very tight versus tight-moderate glycaemic control in pregnant women.	RCT	BWMD: 2	BWMD: 159
**Murphy et al., 2011[49]**	Effect of maternal asthma on increased risk of adverse perinatal outcomes.	Cohort	BWMD: 8<br>LBW: 12	BWMD: 179589<br>LBW: 1109907
**Nabhan and Elsedawy, 2011[50]**	Tight versus very tight control of mild-moderate pre-existing or non-proteinuric gestational hypertension for improving outcomes.	RCT	BWMD: 1	BWMD: 125

RCT: Randomised controlled trials; LBW: Low birthweight; VLBW: Very low birthweight; ELBW: Extremely low birthweight; BWMD: Birthweight mean difference (in grams); NS: Not stated.

References for the meta-analysis papers in the table are listed after the table and are numbered sequentially starting at [1].
Reference numbers for the additional files are separate from the overall reference list for this study.

Table 1.8 Details of meta-analyses included in this study (continued)

**Study**	**Comparison**	**Study design**	**Number of studies by outcome**	**Pooled sample size by outcome**
**Patra et al., 2011[51]**	Effect of maternal alcohol exposure on the risk of low birthweight.	Cohort and case control	LBW: 29	LBW: 277300
**Polyzos et al., 2010[52]**	Effect of treatment of periodontal disease with scaling and root planing during pregnancy on adverse birth outcomes.	RCT	LBW: 8<br>VLBW: 3	LBW: 4929<br>VLBW: 3274
**Pope et al., 2010[53]**	Relationship between indoor air pollution and risk of low birth weight.	Cross-sectional, cohort, case control and RCT	BWMD: 5<br>LBW: 8	BWMD: 13955<br>LBW: NS
**Quinlivan et al., 2011[54]**	Estimated the effect of antenatal dietary interventions used to restrict maternal weight gain (in obese pregnant women) on newborn birth weight.	RCT	BWMD: 4	BWMD: 537
**Raynes-Greenow et al., 2011[55]**	Effect of antibiotic treatment of pregnant women with heavy vaginal urea plasma colonisation on adverse pregnancy outcomes.	RCT	LBW: 1	LBW: 825
**Reveiz et al., 2011[56]**	Compared the effect of Intravenous iron versus regular oral iron on birth outcomes.	RCT	LBW:1<br>BWMD: 3	LBW:100<br>BWMD :237

RCT: Randomised controlled trials; LBW: Low birthweight; VLBW: Very low birthweight; BWMD: Birthweight mean difference (in grams)

References for the meta-analysis papers in the table are listed after the table and are numbered sequentially starting at [1].
Reference numbers for the additional files are separate from the overall reference list for this study.

Table 1.9 Details of meta-analyses included in this study (continued)

Study	Comparison	Study design	Number of studies by outcome	Pooled sample size by outcome
**Rossi and D'Addario, 2011[57]**	Compared the neonatal outcomes of assisted reproductive technique with those of naturally conceived twins.	Cohort	BWMD: 3<br>MLBW: 3<br>VLBW: 3	BWMD: 9793<br>MLBW: 9793<br>VLBW: 9793
**Rumbold et al., 2011[58]**	Any vitamins versus no vitamins (or minimal vitamins) on neonatal outcomes.	RCT	BWMD: 5	BWMD: 7497
**Salmasi et al., 2010[59]**	Effect of environmental tobacco smoke exposure alone without active maternal smoking on perinatal outcomes.	Case-control and Cohort	BWMD: 44<br>LBW: 19	BWMD: 71663<br>LBW: 40790
**Salvig and Lamont, 2011[60]**	Effect of consuming marine n-3 fatty acids during pregnancy on birth outcomes.	RCT	BWMD: 4<br>LBW: 3	BWMD: 1187<br>LBW: 785
**Saraswat et al., 2010[61]**	Perinatal outcomes in women with threatened miscarriage in the first trimester.	Case control and cohort	LBW: 8	LBW: 68897
**Shah 2010[62]**	Risks of adverse pregnancy outcomes among women of different parity.	Cohort	BWMD: 6<br>LBW: 8	BWMD:133533<br>LBW: 2030784
**Shah and Shah, 2010[63]**	Effects of maternal exposure to domestic violence on birth outcomes.	Cohort, cross-sectional and case control	BWMD: 6<br>LBW: 22	BWMD: 17089<br>LBW: 4994431

RCT: Randomised controlled trials; LBW: Low birthweight; VLBW: Very low birthweight; BWMD: Birthweight mean difference (in grams); MLBW: Moderately low birthweight.

References for the meta-analysis papers in the table are listed after the table and are numbered sequentially starting at [1].
Reference numbers for the additional files are separate from the overall reference list for this study.

Table 1.10 Details of meta-analyses included in this study (continued)

Study	Comparison	Study design	Number of studies by outcome	Pooled sample size by outcome
**Shah et al., 2011[64]**	Effect of marital status on birth outcomes.	Cohort and case control	BWMD: 4<br>LBW: 14	BWMD: 240205<br>LBW: 1342583
**Shah et al., 2011[65]**	Pregnancy and neonatal outcomes in Aboriginal women.	Cohort and case control	BWMD: 6<br>LBW: 22<br>Macrosomia: 9	BWMD: 273112<br>LBW: 34,077,737<br>Macrosomia: 5,715,593
**Siegfried et al., 2011[66]**	Effects of antiretroviral treatment (in decreasing the risk of mother-to-child transmission) versus placebo on birth outcomes.	RCT	LBW: 3	LBW: 1305
**Stampalija et al., 2010[67]**	Effects of Uterine artery Doppler ultrasound versus no Doppler ultrasound in 2nd trimester on neonatal outcomes.	RCT	BWMD: 1	BWMD: 3133
**Sturt et al., 2010[68]**	MmaBana (Shapiro 2009)- AZT/3TC/ABC versus AZT/3TC/LPV-r in HIV-Infected Pregnant Women Eligible for Anti-Retroviral Therapy.	RCT	LBW: 1	LBW: 553
**Uppal et al., 2010[69]**	The effect of periodontal disease treatment during pregnancy on the risk of low birth weight.	RCT	LBW: 8	LBW: 5802

RCT: Randomised controlled trials; LBW: Low birthweight; VLBW: Very low birthweight; BWMD: Birthweight mean difference (in grams); Macrosomia: birthweight>4000g

References for the meta-analysis papers in the table are listed after the table and are numbered sequentially starting at [1].
Reference numbers for the additional files are separate from the overall reference list for this study.

Table 1.11 Details of meta-analyses included in this study (continued)

Study	Comparison	Study design	Number of studies by outcome	Pooled sample size by outcome
**van den Broek et al., 2010 [70]**	Effect of vitamin A alone versus placebo or no treatment on newborn outcomes.	RCT	LBW: 3	LBW: 890
**Vazquez and Abalos, 2011[71]***	A.) Outpatient versus inpatient antibiotics (Birthweight (g)).	RCT	BWMD: 1	BWMD: 128
	B.) Cephalosporins once-a-day versus multiple doses (Birthweight< 2500g).	RCT	LBW: 1	LBW: 178
**Veenandaal et al., 2011[72]**	Effects of hyperemesis gravidarum on offspring (LBW).	Cohort and case control	LBW: 5	LBW: 659290
**Wax et al., 2010[73]**	Effects of planned home birth versus planned hospital birth on neonatal outcomes.	Cohort	LBW: 5	LBW: 36701
**Whitworth et al., 2010[74]**	Routine/revealed versus selective/concealed ultrasound in early pregnancy.	RCT	BWMD: 5<br>LBW: 8<br>VLBW: 2	BWMD: 23213<br>LBW: 19337<br>VLBW: 1584
**Widmer et al., 2011[75]**	Effect of single dose versus short-course (7 days) antibiotic for treatment of asymptomatic bacteriuria in pregnancy.	RCT	LBW: 1	LBW: 714
**Wiysonge et al., 2011[76]**	Effect of vitamin A supplementation on risk of mother-to-child transmission of HIV infection.	RCT	BWMD:3<br>LBW:4<br>VLBW⁺: 2	BWMD:1809<br>LBW:2606<br>VLBW⁺:1483

RCT: Randomised controlled trials; LBW: Low birthweight; VLBW: Very low birthweight; BWMD: Birthweight mean difference (in grams); ⁺Very low birthweight defined in this paper as birthweight<2000g; *The meta-analyses are on different interventions.

References for the meta-analysis papers in the table are listed after the table and are numbered sequentially starting at [1].
Reference numbers for the additional files are separate from the overall reference list for this study.

References

1. Abou El Senoun G, Dowswell T, Mousa HA: **Planned home versus hospital care for preterm prelabour rupture of the membranes (PPROM) prior to 37 weeks' gestation.** *Cochrane Database Syst Rev* 2010:CD008053.
2. Akl EA, Gaddam S, Gunukula SK, Honeine R, Jaoude PA, Irani J: **The effects of waterpipe tobacco smoking on health outcomes: a systematic review.** *Int J Epidemiol* 2010, **39:**834-857.
3. Alexander S, Boulvain M, Ceysens G, Haelterman E, Zhang WH: **Repeat digital cervical assessment in pregnancy for identifying women at risk of preterm labour.** *Cochrane Database Syst Rev* 2010:CD005940.
4. Alfirevic Z, Stampalija T, Gyte Gillian ML: **Fetal and umbilical Doppler ultrasound in high-risk pregnancies.** In *Cochrane Database of Systematic Reviews*. Chichester, UK: John Wiley & Sons, Ltd; 2010.
5. Alfirevic Z, Stampalija T, Gyte Gillian ML: **Fetal and umbilical Doppler ultrasound in normal pregnancy.** In *Cochrane Database of Systematic Reviews*. Chichester, UK: John Wiley & Sons, Ltd; 2010.
6. Begley CM, Gyte GM, Murphy DJ, Devane D, McDonald SJ, McGuire W: **Active versus expectant management for women in the third stage of labour.** *Cochrane Database Syst Rev* 2010:CD007412.
7. Bevilacqua E, Brunelli R, Anceschi MM: **Review and meta-analysis: Benefits and risks of multiple courses of antenatal corticosteroids.** *J Matern Fetal Neonatal Med* 2010, **23:**244-260.
8. Blanco CG, Ballesteros AC, Saladich IG, Pla RC: **Glycemic control and pregnancy outcomes in women with type 1 diabetes mellitus using lispro versus regular insulin: A systematic review and meta-analysis.** *Diabetes Technology and Therapeutics* 2011, **13 (9):**907-911.
9. Bonzini M, Palmer KT, Coggon D, Carugno M, Cromi A, Ferrario MM: **Shift work and pregnancy outcomes: a systematic review with meta-analysis of currently available epidemiological studies.** *BJOG* 2011, **118:**1429-1437.
10. Buchanan Sarah L, Crowther Caroline A, Levett Kate M, Middleton P, Morris J: **Planned early birth versus expectant management for women with preterm prelabour rupture of membranes prior to 37 weeks' gestation for improving pregnancy outcome.** In *Cochrane Database of Systematic Reviews*. Chichester, UK: John Wiley & Sons, Ltd; 2010.
11. Buppasiri P, Lumbiganon P, Thinkhamrop J, Ngamjarus C, Laopaiboon M: **Calcium supplementation (other than for preventing or treating hypertension) for improving pregnancy and infant outcomes.** *Cochrane Database Syst Rev* 2011:CD007079.
12. Coleman T, Chamberlain C, Cooper S, Leonardi-Bee J: **Efficacy and safety of nicotine replacement therapy for smoking cessation in pregnancy: systematic review and meta-analysis.** *Addiction (Abingdon, England)* 2011, **106 (1):**52-61.
13. Crowther CA, Han S: **Hospitalisation and bed rest for multiple pregnancy.** *Cochrane Database Syst Rev* 2010:CD000110.
14. Crowther CA, McKinlay CJ, Middleton P, Harding JE: **Repeat doses of prenatal corticosteroids for women at risk of preterm birth for improving neonatal health outcomes.** *Cochrane Database Syst Rev* 2011:CD003935.
15. De-Regil Luz M, Fernández-Gaxiola Ana C, Dowswell T, Peña-Rosas Juan P: **Effects and safety of periconceptional folate supplementation for preventing birth defects.** In *Cochrane Database of Systematic Reviews*. Chichester, UK: John Wiley & Sons, Ltd; 2010.
16. Dhulkotia JS, Ola B, Fraser R, Farrell T: **Oral hypoglycemic agents vs insulin in management of gestational diabetes: a systematic review and metaanalysis.** *Am J Obstet Gynecol* 2010, **203:**457 e451-459.

17. Dodd JM, Grivell RM, Crowther CA, Robinson JS: **Antenatal interventions for overweight or obese pregnant women: a systematic review of randomised trials.** *BJOG* 2010, **117:**1316-1326.
18. Dodd JM, McLeod A, Windrim RC, Kingdom J: **Antithrombotic therapy for improving maternal or infant health outcomes in women considered at risk of placental dysfunction.** *Cochrane Database Syst Rev* 2010**:**CD006780.
19. Dowswell T, Carroli G, Duley L, Gates S, Gülmezoglu AM, Khan-Neelofur D, Piaggio Gilda GP: **Alternative versus standard packages of antenatal care for low-risk pregnancy.** In *Cochrane Database of Systematic Reviews*. Chichester, UK: John Wiley & Sons, Ltd; 2010.
20. Eisele TP, Larsen D, Steketee RW: **Protective efficacy of interventions for preventing malaria mortality in children in Plasmodium falciparum endemic areas.** *Int J Epidemiol* 2010, **39 Suppl 1:**i88-101.
21. Finkelsztejn A, Brooks JB, Paschoal FM, Jr., Fragoso YD: **What can we really tell women with multiple sclerosis regarding pregnancy? A systematic review and meta-analysis of the literature.** *BJOG* 2011, **118:**790-797.
22. Gebreselassie SG, Gashe FE: **A systematic review of effect of prenatal zinc supplementation on birthweight: meta-analysis of 17 randomized controlled trials.** *J Health Popul Nutr* 2011, **29:**134-140.
23. George A, Shamim S, Johnson M, Ajwani S, Bhole S, Blinkhorn A, Ellis S, Andrews K: **Periodontal treatment during pregnancy and birth outcomes: a meta-analysis of randomised trials.** *Int J Evid Based Healthc* 2011, **9:**122-147.
24. Gouin K, Murphy K, Shah PS, Knowledge Synth Grp D: **Effects of cocaine use during pregnancy on low birthweight and preterm birth: systematic review and metaanalyses.** *American Journal of Obstetrics and Gynecology* 2011, **204**.
25. Grellier J, Bennett J, Patelarou E, Smith RB, Toledano MB, Rushton L, Briggs DJ, Nieuwenhuijsen MJ: **Exposure to disinfection by-products, fetal growth, and prematurity: a systematic review and meta-analysis.** *Epidemiology* 2010, **21:**300-313.
26. Grote NK, Bridge JA, Gavin AR, Melville JL, Iyengar S, Katon WJ: **A meta-analysis of depression during pregnancy and the risk of preterm birth, low birth weight, and intrauterine growth restriction.** *Arch Gen Psychiatry* 2010, **67:**1012-1024.
27. Gülmezoglu AM, Azhar M: **Interventions for trichomoniasis in pregnancy.** In *Cochrane Database of Systematic Reviews*. Chichester, UK: John Wiley & Sons, Ltd; 2011.
28. Haider BA, Yakoob MY, Bhutta ZA: **Effect of multiple micronutrient supplementation during pregnancy on maternal and birth outcomes.** *BMC Public Health* 2011, **11 Suppl 3:**S19.
29. Han Z, Lutsiv O, Mulla S, Rosen A, Beyene J, McDonald SD, Knowledge Synth G: **Low gestational weight gain and the risk of preterm birth and low birthweight: a systematic review and meta-analyses.** *Acta Obstetricia Et Gynecologica Scandinavica* 2011, **90:**935-954.
30. Han Z, Mulla S, Beyene J, Liao G, McDonald SD: **Maternal underweight and the risk of preterm birth and low birth weight: a systematic review and meta-analyses.** *Int J Epidemiol* 2011, **40:**65-101.
31. Hodnett ED, Fredericks S, Weston J: **Support during pregnancy for women at increased risk of low birthweight babies.** *Cochrane Database Syst Rev* 2010**:**CD000198.
32. Hofmeyr GJ, Lawrie Theresa A, Atallah Álvaro N, Duley L: **Calcium supplementation during pregnancy for preventing hypertensive disorders and related problems.** In *Cochrane Database of Systematic Reviews*. Chichester, UK: John Wiley & Sons, Ltd; 2010.
33. Imdad A, Bhutta ZA: **Effect of balanced protein energy supplementation during pregnancy on birth outcomes.** *BMC Public Health* 2011, **11 Suppl 3:**S17.
34. Imdad A, Jabeen A, Bhutta ZA: **Role of calcium supplementation during pregnancy in reducing risk of developing gestational hypertensive disorders: a meta-analysis of studies from developing countries.** *BMC Public Health* 2011, **11 Suppl 3:**S18.

35. Kawai K, Spiegelman D, Shankar AH, Fawzi WW: **Maternal multiple micronutrient supplementation and pregnancy outcomes in developing countries: meta-analysis and meta-regression.** *Bull World Health Organ* 2011, **89:**402-411B.
36. Kenyon S, Boulvain M, Neilson James P: **Antibiotics for preterm rupture of membranes.** In *Cochrane Database of Systematic Reviews*. Chichester, UK: John Wiley & Sons, Ltd; 2010.
37. Ladhani NNN, Shah PS, Murphy KE: **Prenatal amphetamine exposure and birth outcomes: A systematic review and metaanalysis.** *American Journal of Obstetrics and Gynecology* 2011, **205 (3):**219.e211-219.e217.
38. Lamont RF, Nhan-Chang CL, Sobel JD, Workowski K, Conde-Agudelo A, Romero R: **Treatment of abnormal vaginal flora in early pregnancy with clindamycin for the prevention of spontaneous preterm birth: a systematic review and metaanalysis.** *Am J Obstet Gynecol* 2011, **205:**177-190.
39. Lassi Zohra S, Haider Batool A, Bhutta Zulfiqar A: **Community-based intervention packages for reducing maternal and neonatal morbidity and mortality and improving neonatal outcomes.** In *Cochrane Database of Systematic Reviews*. Chichester, UK: John Wiley & Sons, Ltd; 2010.
40. Mackeen AD, Seibel-Seamon J, Grimes-Dennis J, Baxter Jason K, Berghella V: **Tocolytics for preterm premature rupture of membranes.** In *Cochrane Database of Systematic Reviews*. Chichester, UK: John Wiley & Sons, Ltd; 2011.
41. Mak A, Cheung MWL, Cheak AAC, Chun-Man Ho R: **Combination of heparin and aspirin is superior to aspirin alone in enhancing live births in patients with recurrent pregnancy loss and positive anti-phospholipid antibodies: A meta-analysis of randomized controlled trials and meta-regression.** *Rheumatology* 2010, **49 (2) (pp 281-288)**.
42. Matevosyan NR: **Periodontal disease and perinatal outcomes.** *Arch Gynecol Obstet* 2011, **283:**675-686.
43. Mathanga DP, Uthman OA, Chinkhumba J: **Intermittent preventive treatment regimens for malaria in HIV-positive pregnant women.** *Cochrane Database Syst Rev* 2011:CD006689.
44. McDonald SD, Han Z, Mulla S, Beyene J, Knowledge Synth G: **Overweight and obesity in mothers and risk of preterm birth and low birth weight infants: systematic review and meta-analyses.** *British Medical Journal* 2010, **341**.
45. McDonald SD, Han Z, Mulla S, Ohlsson A, Beyene J, Murphy KE, Knowledge Synth G: **Preterm birth and low birth weight among in vitro fertilization twins: A systematic review and meta-analyses.** *European Journal of Obstetrics & Gynecology and Reproductive Biology* 2010, **148:**105-113.
46. McLernon DJ, Harrild K, Bergh C, Davies MJ, De Neubourg D, Dumoulin JCM, Gerris J, Kremer JAM, Martikainen H, Mol BW, et al: **Clinical effectiveness of elective single versus double embryo transfer: Meta-analysis of individual patient data from randomised trials.** *BMJ* 2011, **342 (7787):**34.
47. Metcalfe A, Lail P, Ghali WA, Sauve RS: **The association between neighbourhoods and adverse birth outcomes: a systematic review and meta-analysis of multi-level studies.** *Paediatr Perinat Epidemiol* 2011, **25:**236-245.
48. Middleton P, Crowther Caroline A, Simmonds L, Muller P: **Different intensities of glycaemic control for pregnant women with pre-existing diabetes.** In *Cochrane Database of Systematic Reviews*. Chichester, UK: John Wiley & Sons, Ltd; 2010.
49. Murphy VE, Namazy JA, Powell H, Schatz M, Chambers C, Attia J, Gibson PG: **A meta-analysis of adverse perinatal outcomes in women with asthma.** *BJOG* 2011, **118:**1314-1323.
50. Nabhan AF, Elsedawy MM: **Tight control of mild-moderate pre-existing or non-proteinuric gestational hypertension.** *Cochrane Database Syst Rev* 2011:CD006907.

51. Patra J, Bakker R, Irving H, Jaddoe VWV, Malini S, Rehm J: **Dose-response relationship between alcohol consumption before and during pregnancy and the risks of low birthweight, preterm birth and small for gestational age (SGA)-a systematic review and meta-analyses.** *Bjog-an International Journal of Obstetrics and Gynaecology* 2011, **118:**1411-1421.
52. Polyzos NP, Polyzos IP, Zavos A, Valachis A, Mauri D, Papanikolaou EG, Tzioras S, Weber D, Messinis IE: **Obstetric outcomes after treatment of periodontal disease during pregnancy: systematic review and meta-analysis.** *BMJ* 2010, **341:**c7017.
53. Pope DP, Mishra V, Thompson L, Siddiqui AR, Rehfuess EA, Weber M, Bruce NG: **Risk of low birth weight and stillbirth associated with indoor air pollution from solid fuel use in developing countries.** *Epidemiol Rev* 2010, **32:**70-81.
54. Quinlivan JA, Julania S, Lam L: **Antenatal dietary interventions in obese pregnant women to restrict gestational weight gain to institute of medicine recommendations: A meta-analysis.** *Obstetrics and Gynecology* 2011, **118 (6):**1395-1401.
55. Raynes-Greenow Camille H, Roberts Christine L, Bell Jane C, Peat B, Gilbert Gwendolyn L, Parker S: **Antibiotics for ureaplasma in the vagina in pregnancy.** In *Cochrane Database of Systematic Reviews*. Chichester, UK: John Wiley & Sons, Ltd; 2011.
56. Reveiz L, Gyte GM, Cuervo LG, Casasbuenas A: **Treatments for iron-deficiency anaemia in pregnancy.** *Cochrane Database Syst Rev* 2011**:**CD003094.
57. Rossi AC, D'Addario V: **Neonatal outcomes of assisted and naturally conceived twins: systematic review and meta-analysis.** *J Perinat Med* 2011, **39:**489-493.
58. Rumbold A, Middleton P, Pan N, Crowther Caroline A: **Vitamin supplementation for preventing miscarriage.** In *Cochrane Database of Systematic Reviews*. Chichester, UK: John Wiley & Sons, Ltd; 2011.
59. Salmasi G, Grady R, Jones J, McDonald SD: **Environmental tobacco smoke exposure and perinatal outcomes: a systematic review and meta-analyses.** *Acta Obstet Gynecol Scand* 2010, **89:**423-441.
60. Salvig JD, Lamont RF: **Evidence regarding an effect of marine n-3 fatty acids on preterm birth: a systematic review and meta-analysis.** *Acta Obstet Gynecol Scand* 2011, **90:**825-838.
61. Saraswat L, Bhattacharya S, Maheshwari A: **Maternal and perinatal outcome in women with threatened miscarriage in the first trimester: a systematic review.** *BJOG* 2010, **117:**245-257.
62. Shah PS: **Parity and low birth weight and preterm birth: a systematic review and meta-analyses.** *Acta Obstet Gynecol Scand* 2010, **89:**862-875.
63. Shah PS, Shah J: **Maternal exposure to domestic violence and pregnancy and birth outcomes: a systematic review and meta-analyses.** *J Womens Health (Larchmt)* 2010, **19:**2017-2031.
64. Shah PS, Zao J, Ali S: **Maternal marital status and birth outcomes: a systematic review and meta-analyses.** *Matern Child Health J* 2011, **15:**1097-1109.
65. Shah PS, Zao J, Al-Wassia H, Shah V: **Pregnancy and neonatal outcomes of aboriginal women: a systematic review and meta-analysis.** *Womens Health Issues* 2011, **21:**28-39.
66. Siegfried N, van der Merwe L, Brocklehurst P, Sint Tin T: **Antiretrovirals for reducing the risk of mother-to-child transmission of HIV infection.** In *Cochrane Database of Systematic Reviews*. Chichester, UK: John Wiley & Sons, Ltd; 2011.
67. Stampalija T, Gyte Gillian ML, Alfirevic Z: **Utero-placental Doppler ultrasound for improving pregnancy outcome.** In *Cochrane Database of Systematic Reviews*. Chichester, UK: John Wiley & Sons, Ltd; 2010.
68. Sturt AS, Dokubo EK, Sint TT: **Antiretroviral therapy (ART) for treating HIV infection in ART-eligible pregnant women.** *Cochrane Database Syst Rev* 2010**:**CD008440.

69. Uppal A, Uppal S, Pinto A, Dutta M, Shrivatsa S, Dandolu V, Mupparapu M: **The effectiveness of periodontal disease treatment during pregnancy in reducing the risk of experiencing preterm birth and low birth weight: a meta-analysis.** *J Am Dent Assoc* 2010, **141:**1423-1434.
70. van den Broek N, Dou L, Othman M, Neilson JP, Gates S, Gulmezoglu AM: **Vitamin A supplementation during pregnancy for maternal and newborn outcomes.** *Cochrane Database Syst Rev* 2010**:**CD008666.
71. Vazquez JC, Abalos E: **Treatments for symptomatic urinary tract infections during pregnancy.** *Cochrane Database Syst Rev* 2011**:**CD002256.
72. Veenendaal MVE, Van Abeelen AFM, Painter RC, Van Der Post JAM, Roseboom T: **Consequences of hyperemesis gravidarum for offspring: A systematic review and meta-analysis.** *BJOG: An International Journal of Obstetrics and Gynaecology* 2011, **118 (11):**1302-1313.
73. Wax JR, Lucas FL, Lamont M, Pinette MG, Cartin A, Blackstone J: **Maternal and newborn outcomes in planned home birth vs planned hospital births: a metaanalysis.** *Am J Obstet Gynecol* 2010, **203:**243 e241-248.
74. Whitworth M, Bricker L, Neilson James P, Dowswell T: **Ultrasound for fetal assessment in early pregnancy.** In *Cochrane Database of Systematic Reviews*. Chichester, UK: John Wiley & Sons, Ltd; 2010.
75. Widmer M, Gülmezoglu AM, Mignini L, Roganti A: **Duration of treatment for asymptomatic bacteriuria during pregnancy.** In *Cochrane Database of Systematic Reviews*. Chichester, UK: John Wiley & Sons, Ltd; 2011.
76. Wiysonge CS, Shey M, Kongnyuy EJ, Sterne JA, Brocklehurst P: **Vitamin A supplementation for reducing the risk of mother-to-child transmission of HIV infection.** *Cochrane Database Syst Rev* 2011**:**CD003648.
