# Supplementary material for: Dichotomisation of a continuous outcome and effect on meta-analyses: illustration of the distributional approach using the outcome birthweight
Source: Syst Rev. 2014 Jun 12;3:63. doi: 10.1186/2046-4053-3-63 (PMC4063432; doi:10.1186/2046-4053-3-63)
Supplement: Additional file 2: Table S1 — Meta-analyses for which secondary analyses could not be performed with reasons. [file 2046-4053-3-63-S2.pdf]

Additional file 2

Table 1 Meta-analyses for which secondary analyses could not be performed with reasons

Meta-analysis	Reason
Han 2011[1]	Details of primary studies included in mean birthweight outcome were unclear
Han 2011[2]	Details of primary studies included in mean birthweight outcome were not reported
Mcdonald 2010[3]	Details of primary studies included in mean birthweight outcome were not reported
Mclernon 2010[4]	Details of individual patient data included in meta-analyses could not be accessed
Rossi 2010 [5]	Data parameters of primary studies included in mean birthweight outcome were unclear
Shah 2010[6]	Details of primary studies included in mean birthweight outcome were not reported
Shah 2010[7]	Details of primary studies are unclear
Shah 2011[8]	Details of primary studies are unclear
Shah 2011[9]	Details of primary studies are unclear

References for the meta-analysis papers in the table are listed after the table and are numbered sequentially starting at [1]. Reference numbers for the additional files are separate from the overall reference list for this study.

References

1. Han Z, Lutsiv O, Mulla S, Rosen A, Beyene J, McDonald SD, Knowledge Synth G: **Low gestational weight gain and the risk of preterm birth and low birthweight: a systematic review and meta-analyses.** *Acta Obstetricia Et Gynecologica Scandinavica* 2011, **90:**935-954.
2. Han Z, Mulla S, Beyene J, Liao G, McDonald SD: **Maternal underweight and the risk of preterm birth and low birth weight: a systematic review and meta-analyses.** *Int J Epidemiol* 2011, **40:**65-101.
3. McDonald SD, Han Z, Mulla S, Beyene J, Knowledge Synth G: **Overweight and obesity in mothers and risk of preterm birth and low birth weight infants: systematic review and meta-analyses.** *British Medical Journal* 2010, **341**.
4. McLernon DJ, Harrild K, Bergh C, Davies MJ, De Neubourg D, Dumoulin JCM, Gerris J, Kremer JAM, Martikainen H, Mol BW, et al: **Clinical effectiveness of elective single versus double embryo transfer: Meta-analysis of individual patient data from randomised trials.** *BMJ* 2011, **342 (7787):**34.
5. Rossi AC, D'Addario V: **Neonatal outcomes of assisted and naturally conceived twins: systematic review and meta-analysis.** *J Perinat Med* 2011, **39:**489-493.
6. Shah PS: **Parity and low birth weight and preterm birth: a systematic review and meta-analyses.** *Acta Obstet Gynecol Scand* 2010, **89:**862-875.
7. Shah PS, Shah J: **Maternal exposure to domestic violence and pregnancy and birth outcomes: a systematic review and meta-analyses.** *J Womens Health (Larchmt)* 2010, **19:**2017-2031.
8. Shah PS, Zao J, Ali S: **Maternal marital status and birth outcomes: a systematic review and meta-analyses.** *Matern Child Health J* 2011, **15:**1097-1109.
9. Shah PS, Zao J, Al-Wassia H, Shah V: **Pregnancy and neonatal outcomes of aboriginal women: a systematic review and meta-analysis.** *Womens Health Issues* 2011, **21:**28-39.
